# Supplementary material for: Mechanism-centric regulatory network identifies NME2 and MYC programs as markers of Enzalutamide resistance in CRPC
Source: Nat Commun. 2024 Jan 8;15:352. doi: 10.1038/s41467-024-44686-5 (PMC10774320; doi:10.1038/s41467-024-44686-5)
Supplement: Supplementary file 1 — Supplementary Information [file 41467_2024_44686_MOESM1_ESM.pdf]

Mechanism-centric regulatory network identifies NME2 and MYC programs as markers of Enzalutamide resistance in CRPC

Panja, S. et al

Supplementary Materials

SUPPLEMENTARY FIGURES:

Supplementary Fig. 1 (Related to Fig. 1). AR expression and activity are higher in Enzalutamide-resistant phenotypes.

Supplementary Fig. 2 (Related to Fig. 2). Bootstrap consistency is confirmed by the similarity of significant edge distributions across bootstrap runs.

Supplementary Fig. 3 (Related to Fig. 3). Mechanism-centric network mining identifies molecular pathways and TR programs that govern progression to Enzalutamide resistance.

Supplementary Fig. 4(Related to Fig. 4). VIF analysis identifies multi-collinearity between the transcriptional regulatory programs affecting MYC pathway.

Supplementary Fig. 5 (Related to Fig. 4). Activity levels of NME2 transcriptional program and MYC molecular pathway reveal significant changes across Enzalutamide-related conditions.

Supplementary Fig. 6 (Related to Fig. 5). Upregulation of AR, CK8, and CD45 identify adenocarcinoma prostate cancer cells in pre- and post-Enzalutamide conditions.

Supplementary Fig. 7 (Related to Fig. 5). Association between NME2 TR and MYC pathway activities demonstrates their correlation across multiple CRPC cohorts.

Supplementary Fig. 8 (Related to Fig. 5). Kaplan-Meier survival analysis and ROC analysis on Abiraterone-specific cohorts demonstrate that NME2 and MYC are Enzalutamide specific.

Supplementary Fig. 9 (Related to Fig. 5). Comparative analysis to different computational methods demonstrates superiority of TR-2-PATH approach.

Supplementary Fig. 10 (Related to Fig. 7). MYC inhibition reduces viability and colony formation in Enzalutamide-resistant conditions.

Supplementary Fig. 11 (Related to Fig. 7). MYCi975 degrades MYC in C42B-EnzaRes cells**.**

Supplementary Fig. 12 (Related to Fig. 7). Validation of NME2 CRISPR knockout in C42B-EnzaRes cells.

Supplementary Fig. 13 (Related to Fig. 7). Individual tumor growth curves and mouse body weight plots for in vivo study.

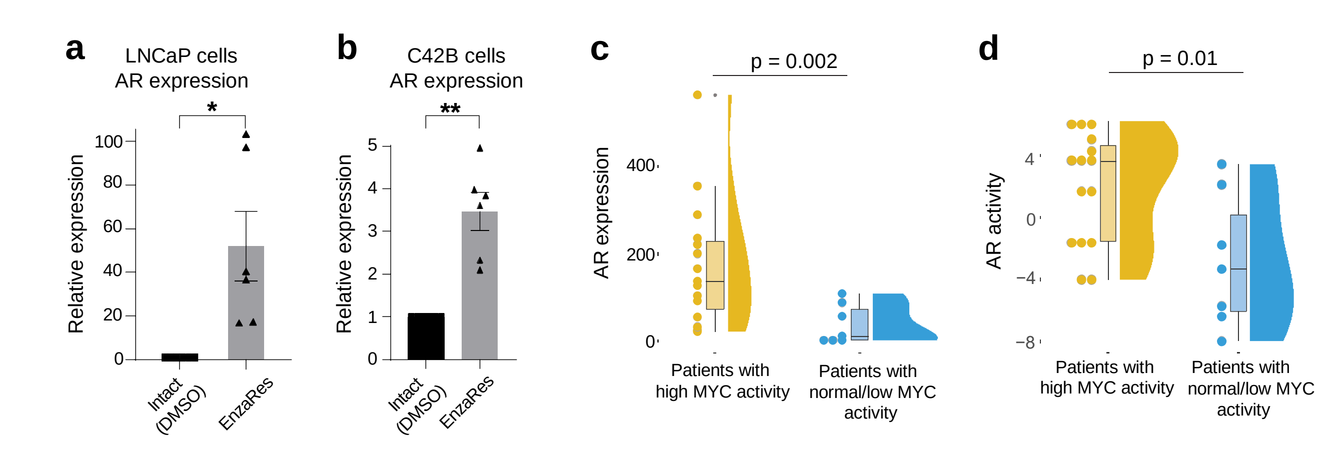

Supplementary Fig. 1 (Related to Fig. 1). AR expression and activity are higher in Enzalutamide-resistant phenotypes. (a-b) AR expression in Intact (treated with DMSO) and Enzalutamide-resistant (EnzaRes) **(a)** LNCaP and **(b)** C42B cell lines, as shown using qRT-PCR. Data are presented as mean values +/- SEM from 6 independent biological replicates. Source data are provided as a Source Data file. **(c)** Comparing AR expression and **(d)** AR activity in CRPC patients from the Abida et al. patient cohort (n = 22) with high MYC activities (yellow) and normal/low MYC activities (blue). P-values were estimated using a one-tailed Welch t-test. * p-value < 0.05, *** p-value ≤ 0.001. In boxplots, the center line corresponds to the median and the box limits correspond to the first and third quartiles (the 25th and 75th percentiles). The upper and lower whiskers extend to the maximum or minimum value within 1.5 times the interquartile range, respectively. Source data are provided as a Source Data file.

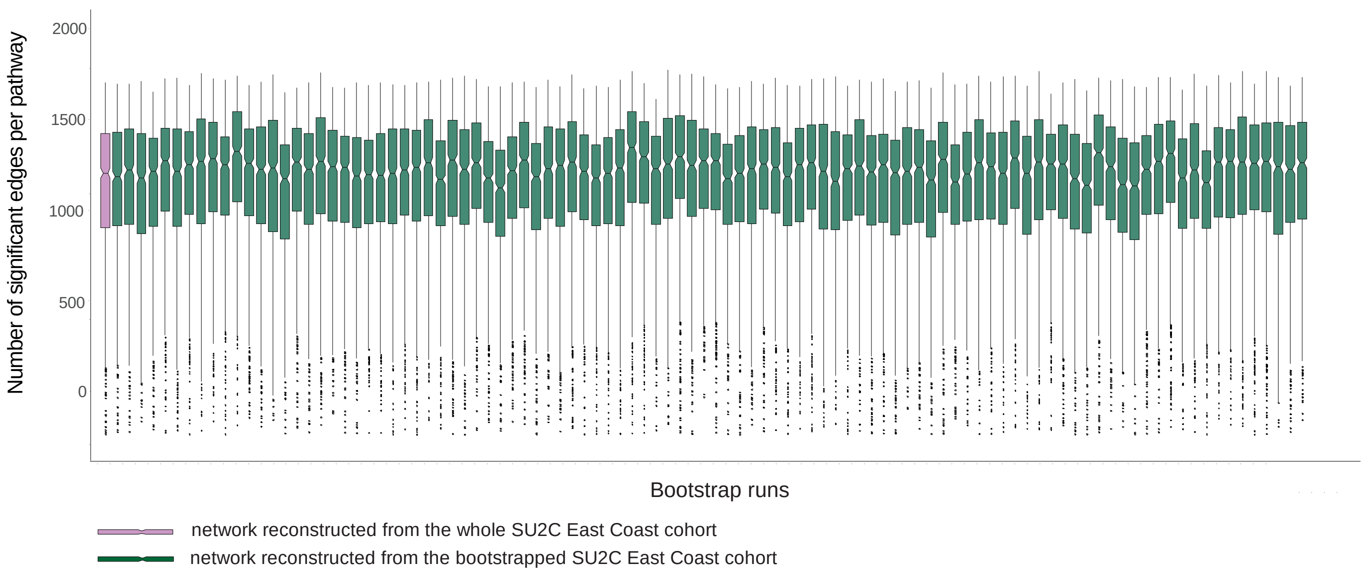

Supplementary Fig. 2 (Related to Fig. 2). Bootstrap consistency is confirmed by the similarity of significant edge distributions across bootstrap runs. The consistency of bootstrap runs in the SU2C East Coast cohort is demonstrated through comparison of the distribution of number of significant edges for each pathway across runs. Pink denotes the result from the original (whole) SU2C East Coast cohort and green denotes results from the bootstrap runs on the same cohort. In boxplots, the center line corresponds to the median and the box limits correspond to the first and third quartiles (the 25th and 75th percentiles). The upper and lower whiskers extend to the maximum or minimum value within 1.5 times the interquartile range, respectively. Source data are provided as a Source Data file.

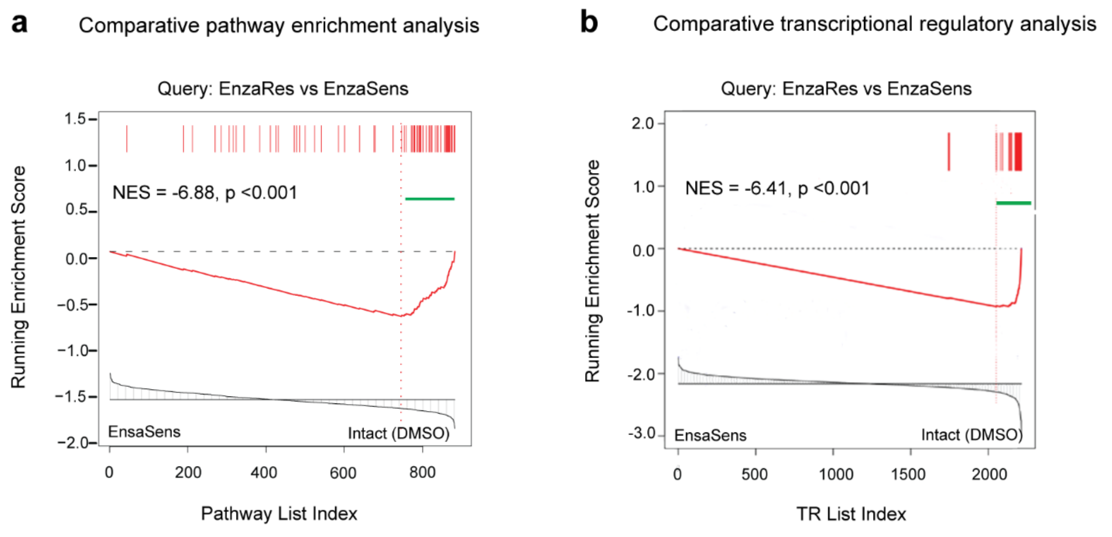

Supplementary Fig. 3 (Related to Fig. 3). Mechanism-centric network mining identifies molecular pathways and TR programs that govern progression to Enzalutamide resistance. **(a)** GSEA performed on pathway activity levels between a reference signature comparing Enzalutamide-sensitive (EnzaSens, n = 4) to Intact (treated with DMSO, n = 4) samples and query signature comparing Enzalutamide-resistant (EnzaRes, n = 4) and Enzalutamide-sensitive (EnzaSens, n = 4) samples (query set was defined as up-regulated pathways at p-value < 0.05). GSEA NES (normalized enrichment score) and p-value were estimated using 1,000 pathway permutations in the reference signature. Source data are provided in Supplementary Data 4C-D **(b)** GSEA performed on TR activity levels between a reference signature comparing Enzalutamide-sensitive (EnzaSens, n = 4) to Intact (treated with DMSO, n = 4) samples and query signature comparing Enzalutamide-resistant (EnzaRes, n = 4) and Enzalutamide-sensitive (EnzaSens, n = 4) samples (query set was defined as up-regulated TRs at p-value < 0.05). NES (normalized enrichment score) and p-value were estimated using 1,000 TR permutations in the reference signature. Source data are provided in Supplementary Data 4E-F.

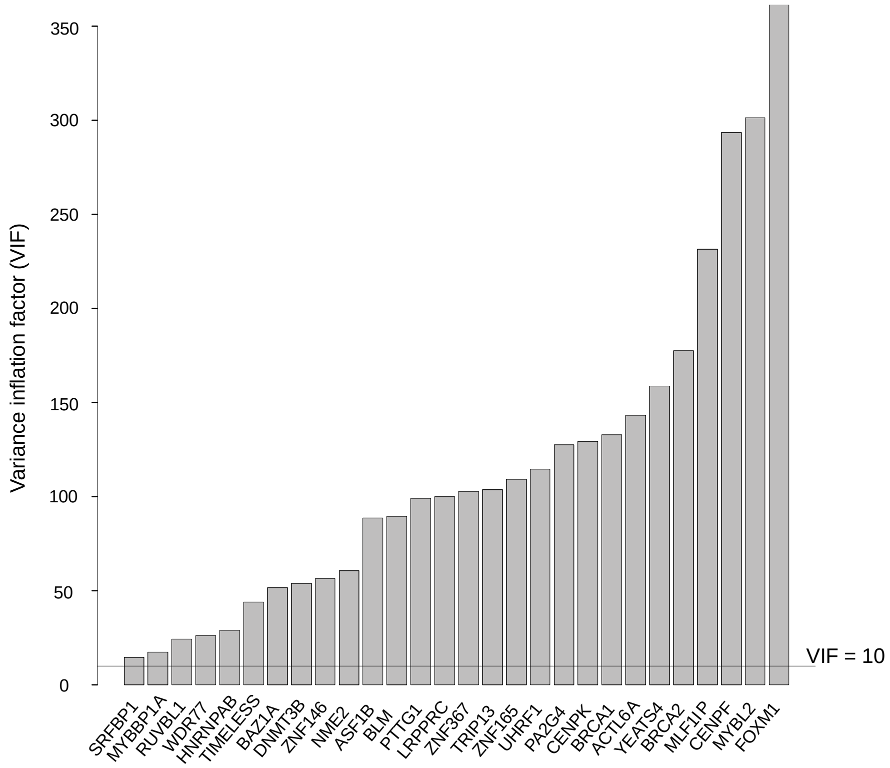

Supplementary Fig. 4 (Related to Fig. 4). VIF analysis identifies multi-collinearity between the transcriptional regulatory programs affecting MYC pathway. Bar plot representation of VIF (variance inflation factor) analysis. Each bar corresponds to VIF value for the indicated transcriptional regulatory program (shown on x-axis). Source data are provided as a Source Data file.

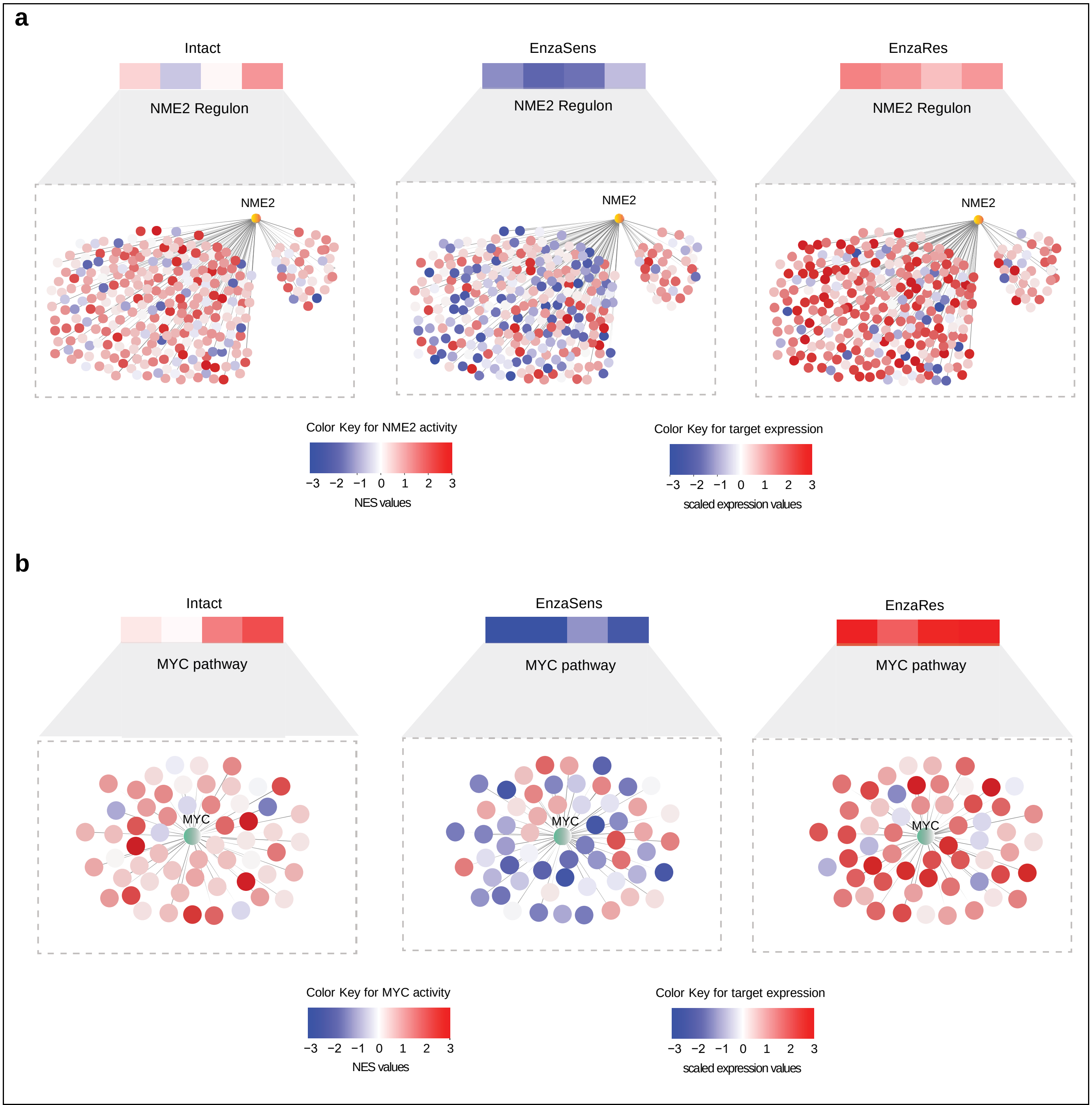

Supplementary Fig. 5 (Related to Fig. 4). Activity levels of NME2 transcriptional program and MYC molecular pathway reveal significant changes across Enzalutamide-related conditions. (a) Expression levels of NME2 activated targets (n = 368) across Intact (treated with DMSO), Enza-sensitive (i.e., EnzaSens), and Enza-resistant (i.e., EnzaRes) phenotypes. **(b)** Expression levels of MYC pathway genes (n = 58) across Intact (treated with DMSO), Enza-sensitive (i.e., EnzaSens), and Enza-resistant (i.e., EnzaRes) phenotypes. Red denotes over-expression and blue denotes under-expression. Source data are provided as a Source Data file.

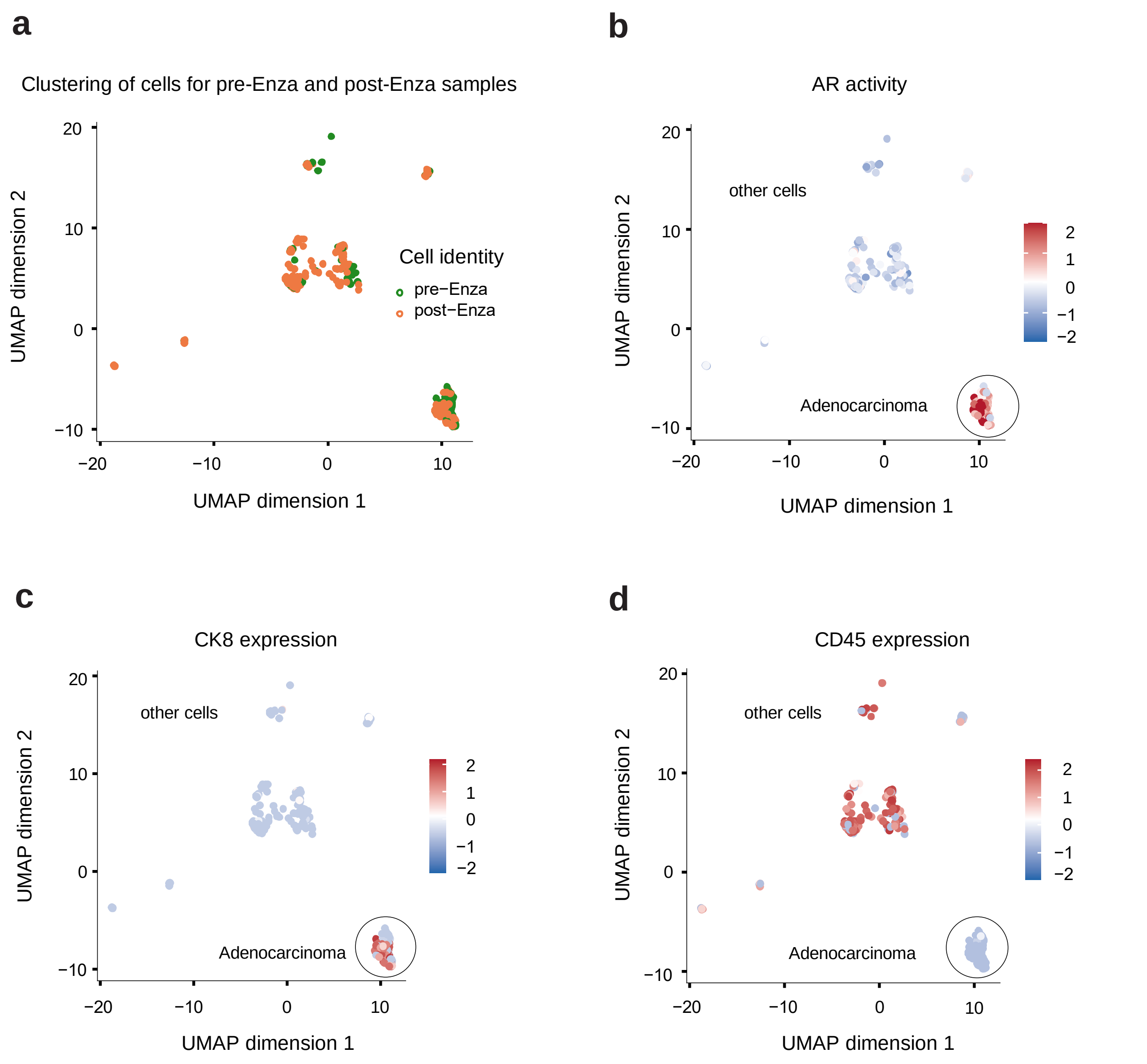

Supplementary Fig. 6 (Related to Fig. 5). Upregulation of AR, CK8, and CD45 identify adenocarcinoma prostate cancer cells in pre- and post-Enzalutamide conditions. UMAP representation of **(a)** pre- and post-Enza cell populations, **(b)** AR activity levels (red denotes higher activity and blue denotes lower activity), **(c)** CK8 expression levels (red denotes over-expression and blue denotes under-expression), and **(d)** CD45 expression levels (red denotes over-expression and blue denotes to under-expression), identifying a group of adenocarcinoma prostate cells (circle). Source data are provided as a Source Data file.

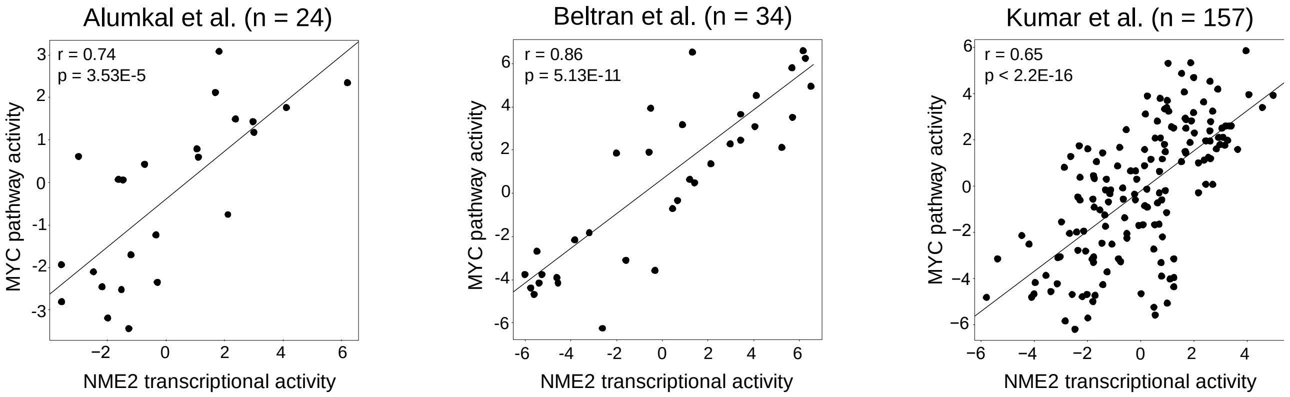

Supplementary Fig. 7 (Related to Fig. 5). Comparative analysis between NME2 TR and MYC pathway activities demonstrates their correlation across multiple CRPC cohorts. Pearson correlation analysis between NME2 TR activity and MYC pathway activity in Alumkal et al. (n = 24), Beltran et al. (CRPC samples, n = 34), and Kumar et al. (n = 157) CRPC cohorts. Pearson r and p-values are indicated. Source data are provided as a Source Data file.

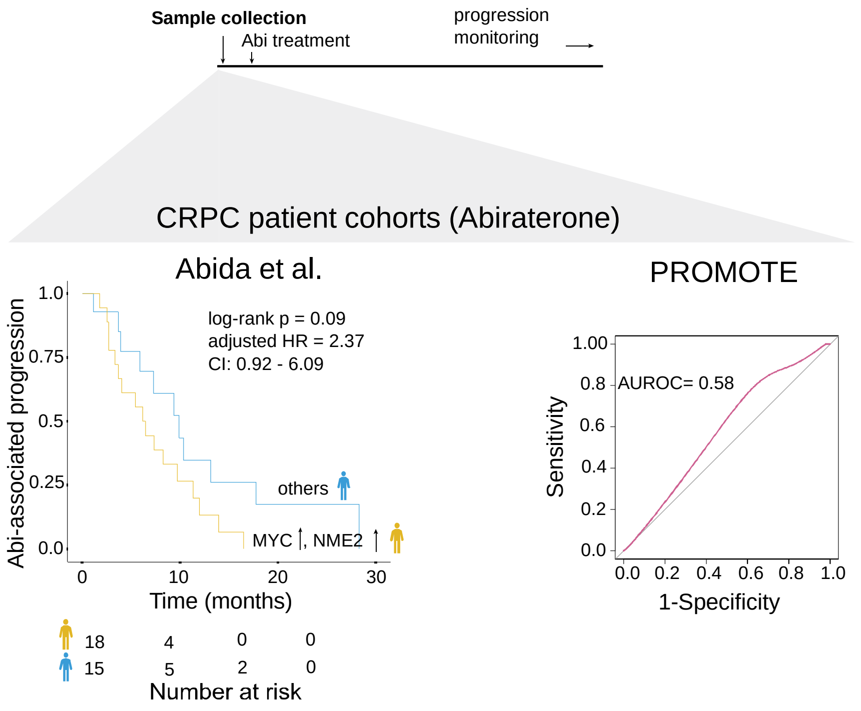

Supplementary Fig. 8 (Related to Fig. 5). Kaplan-Meier survival analysis and ROC analysis on Abiraterone-specific cohorts demonstrate that NME2 and MYC are Enzalutamide specific. (Left) Kaplan-Meier survival analysis, comparing high-MYC and high-NME2 group (yellow) to the rest of the patients (blue) in Abida et al. cohort subjected to adjuvant Abiraterone (n = 33). Log-rank p-value, adjusted HR, and CI are indicated. **(Right)** ROC analysis using NME2 TR and MYC pathway activity levels to compare CRPC patients with poor and favorable Abiraterone response from PROMOTE cohort (n = 77). AUROC (area under ROC) is indicated. Source data are provided as a Source Data file.

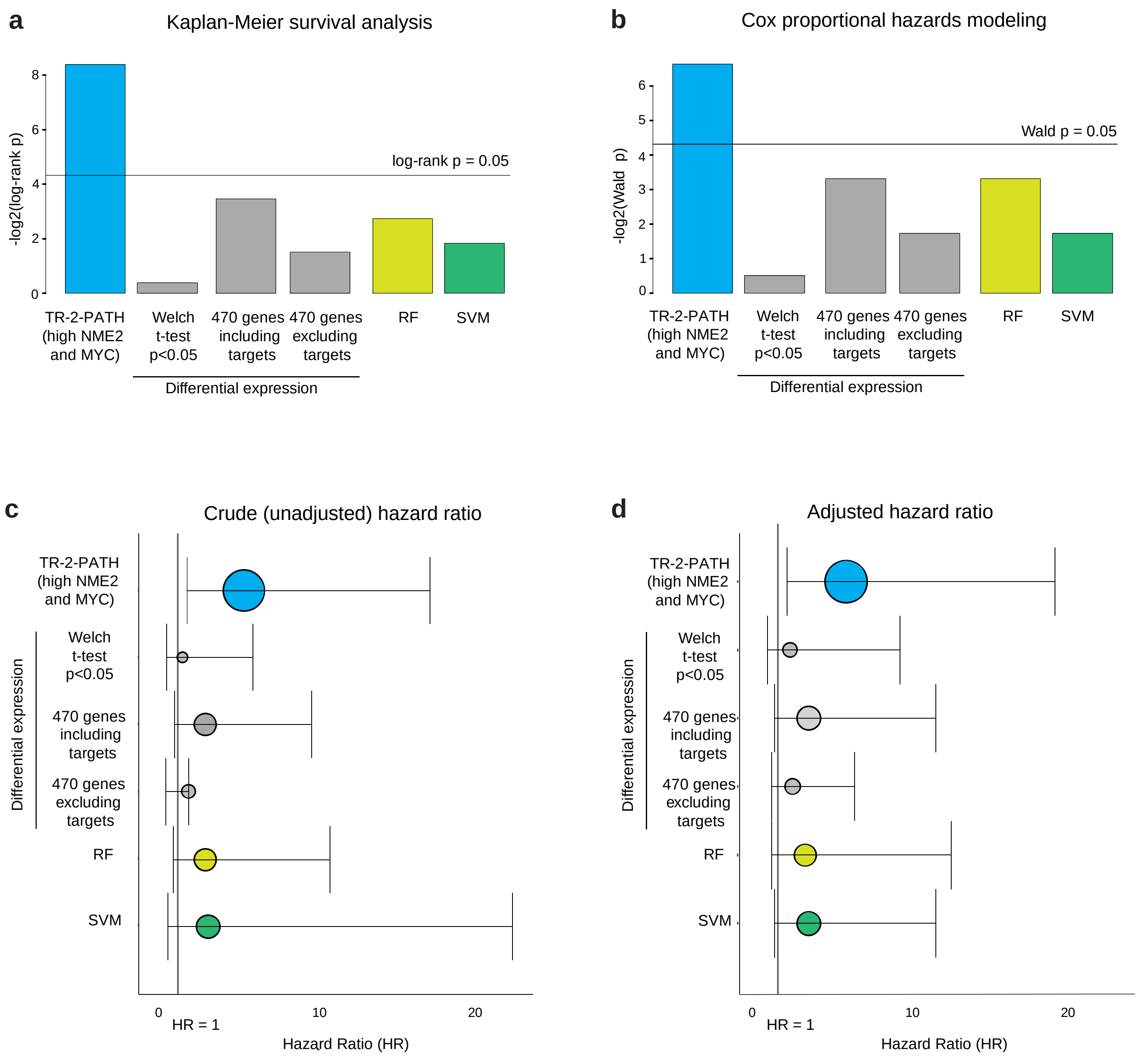

Supplementary Fig. 9 (Related to Fig. 5). Comparative analysis to different computational methods demonstrates superiority of TR-2-PATH approach. (a-d) Comparison of different methods with respect to their ability to predict Enzalutamide resistance. Methods include TR-2-PATH (high-MYC and high-NME2), differential expression analysis between Intact, EnzaSens and EnzaRes phenotypes (Welch t-test p-value <0.05, top 470 genes *including* NME2 TR targets and MYC pathway genes, top 470 genes *excluding* NME2 TR targets and MYC pathway genes), top 10 predictions from Random (survival) Forests (RF) method, and top 10 predictions from Support Vector Machine (SVM) method. Comparison among methods was done using: **(a)** Kaplan-Meier survival analysis (log-rank p-value indicated), **(b)** Cox modeling (Wald p-value indicated), **(c)** crude (unadjusted) hazards ratio, and **(d)** adjusted (for age at diagnosis and Gleason score) hazards ratio. Circles correspond to hazard ratio values and whiskers to their Confidence Intervals, HR=1 is indicated as a vertical line in **(c-d)**. Source data are provided as a Source Data file.

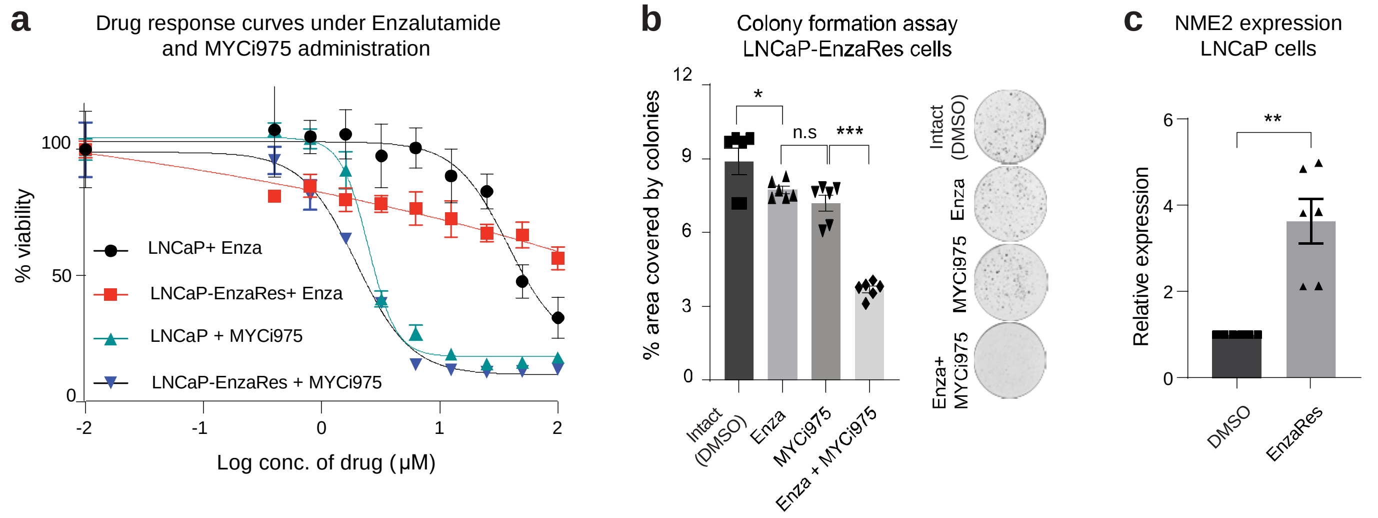

Supplementary Fig. 10 (Related to Fig. 7). MYC inhibition reduces viability and colony formation in Enzalutamide-resistant conditions. (a) Drug response curves of Enzalutamide-naïve LNCaP or Enzalutamide-resistant LNCaP cells treated with Enzalutamide and/or MYCi975. **(b)** Colony formation assay using Enzalutamide resistant LNCaP cells (LNCaP-EnzaRes) in Intact (treated with DMSO), treated with Enza (10µM), MYCi975 (2µM) or a combination of Enza+MYCi975 (10µM+2µM. Cells were grown in the presence of respective drugs. Data are presented as mean values +/- SEM from 6 independent biological replicates, indicating quantification of Crystal Violet trapped by migrated cells. P-value is estimated utilizing one-tailed Welch t-test. * p-value < 0.05, *** p-value ≤ 0.001. **(c)** Expression of NME2 in Intact (DMSO) and Enzalutamide-resistant (EnzaRes) LNCaP cells, using qRT-PCR, data are presented as mean values +/- SEM from 6 independent biological replicates. P-value is estimated using one-tailed Welch t-test. ** p-value ≤ 0.01. Source data for all the panels in this figure are provided as a Source Data file.

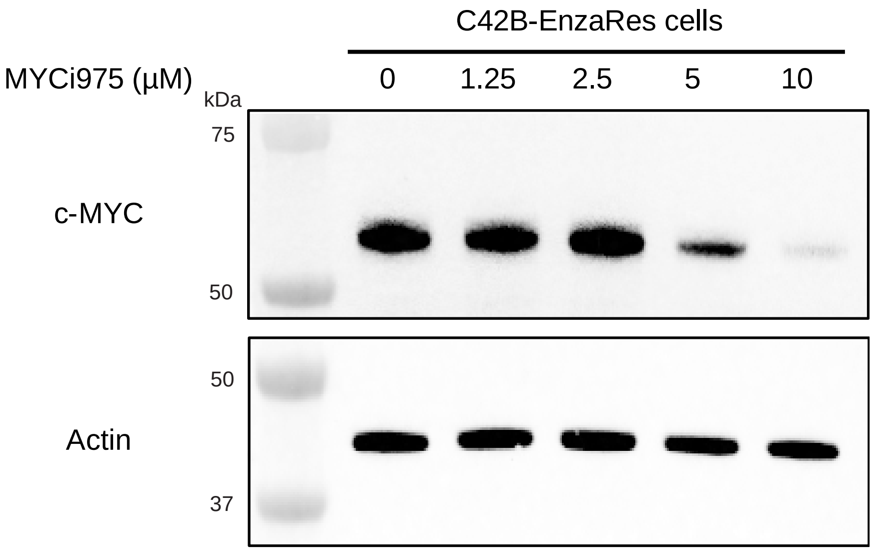

Supplementary Fig. 11 (Related to Fig. 7). MYCi975 degrades MYC in C42B-EnzaRes cells. Western blot of c-MYC and Actin protein levels in C42B-EnzaRes cells after treatment with MYCi975 for 24 hours at indicated concentrations. (n = 3 biologically independent experiments, representative blot shown). Uncropped blots are provided below.

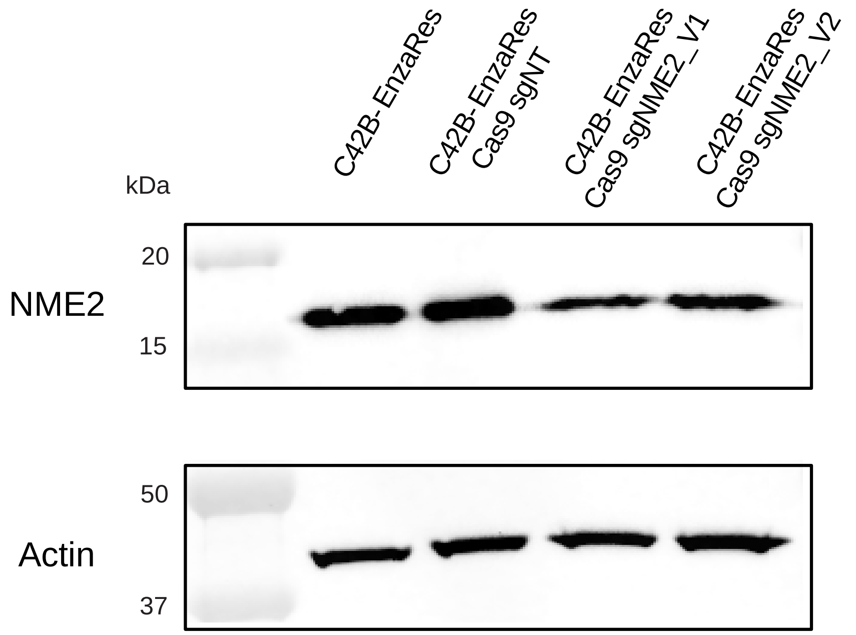

Supplementary Fig. 12 (Related to Fig. 7). Validation of NME2 CRISPR knockout in C42B-EnzaRes cells. Western blot of NME2 and GAPDH protein levels in C42B-EnzaRes cells after performing CRISPR KO with two different sgRNAs targeting NME2, inserted in the pLentiCRISPR-v2 lentiviral plasmid (n = 3 biologically independent experiments, representative blot shown). Uncropped blots are provided below.

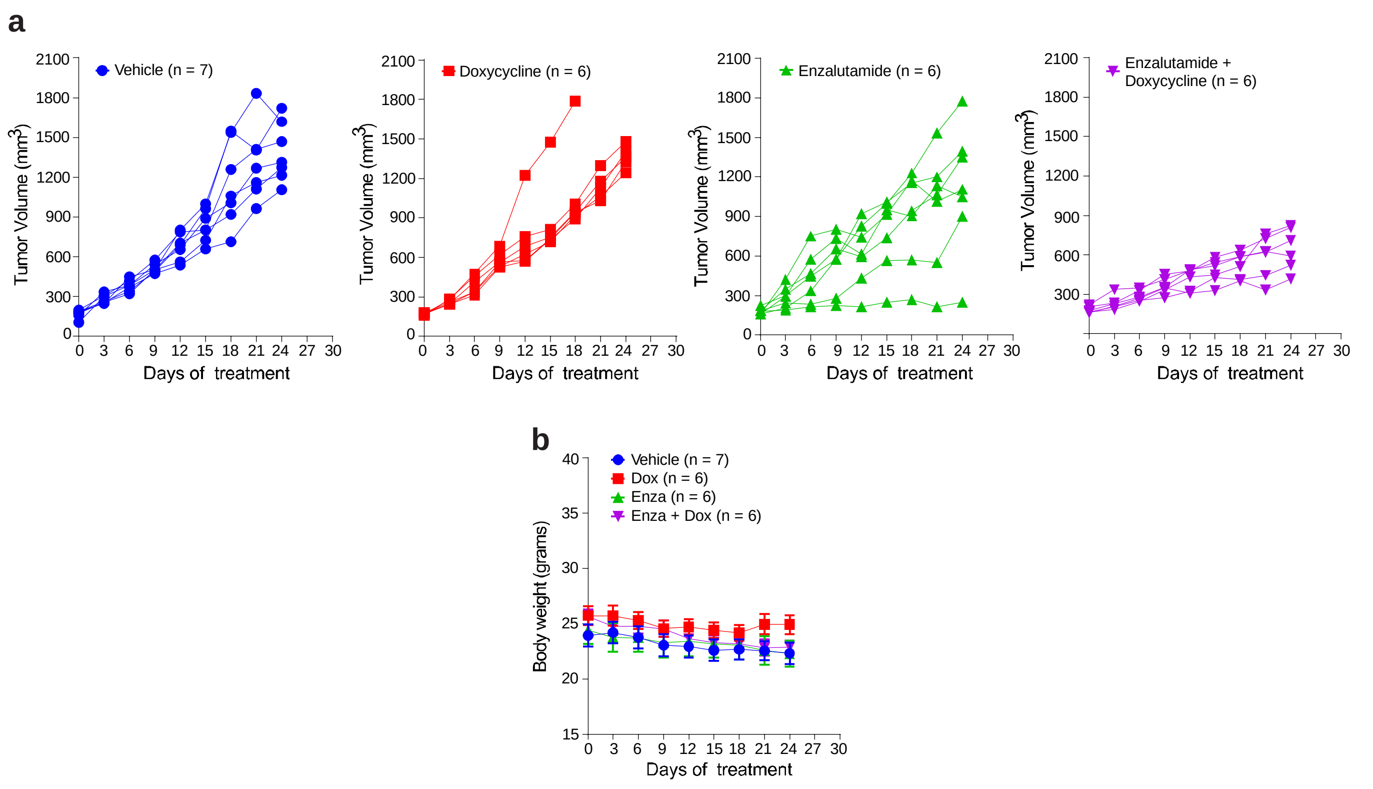

Supplementary Fig. 13 (Related to Fig. 7). Individual tumor growth curves and mouse body weight plots for in vivo study. (a) Tumor growth curves over duration of treatment for individual mice from each treatment arm from the study shown in Fig. 7I **(b)** Mouse average body weight over the duration of treatment from the study shown in Fig. 7I. Source data for all the panels in this figure are provided as a Source Data file.

Uncropped blots for Supplemental Fig. 11

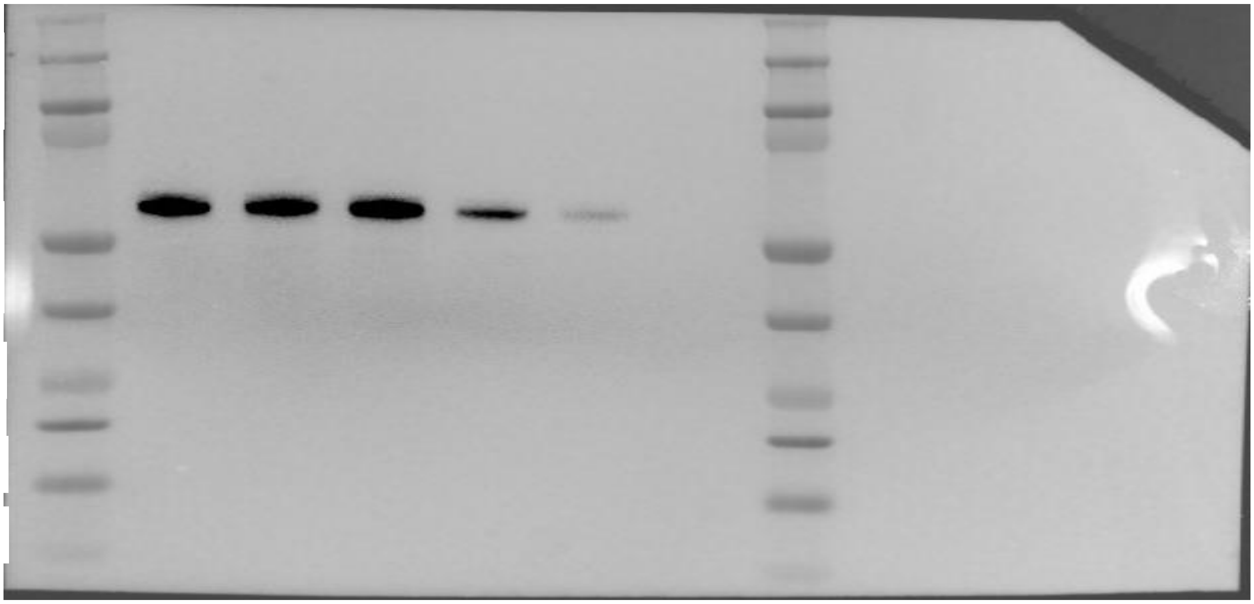

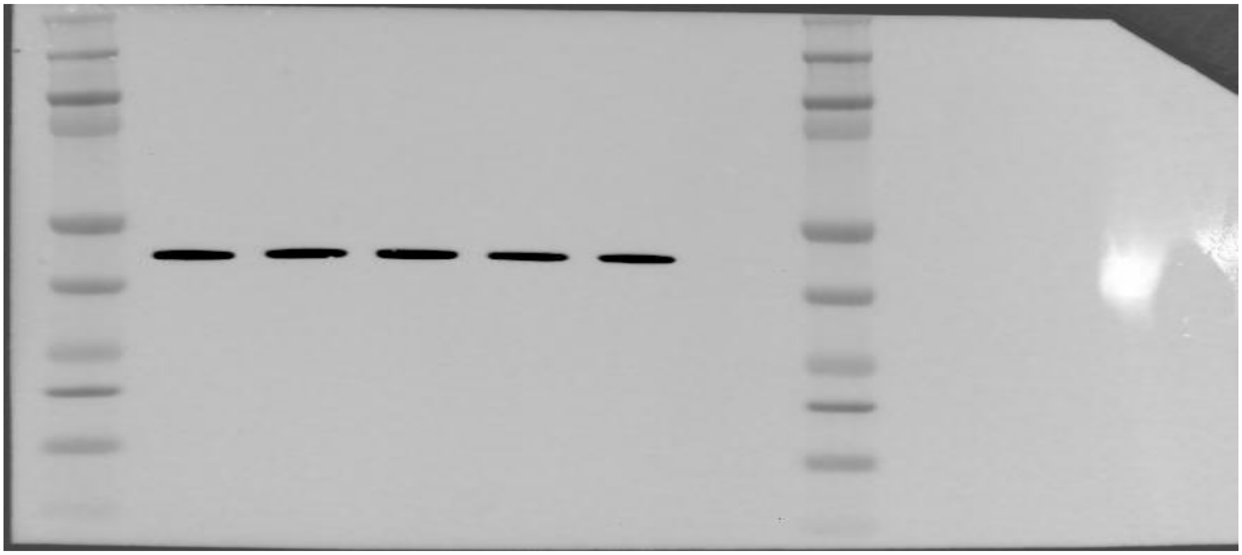

Uncropped blots for Supplemental Fig. 12

NME2

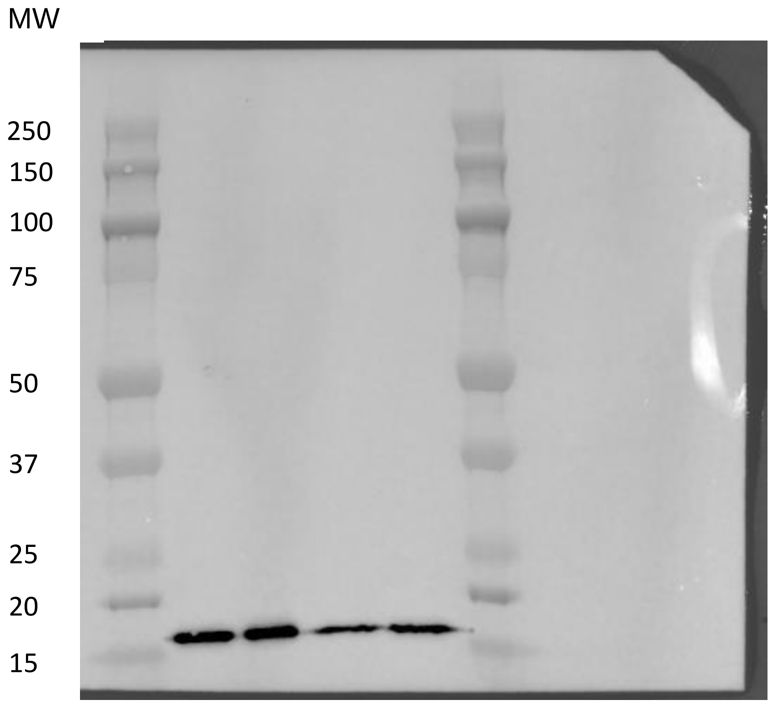

Actin

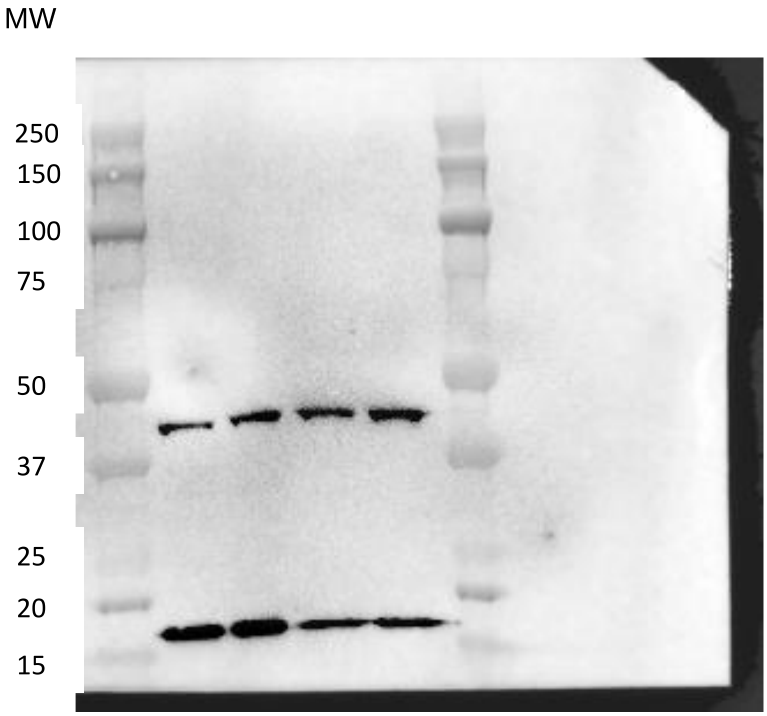

Uncropped blots for Fig. 7h

NME2

MW
250
150
100
75
50
37
25
20
15
10

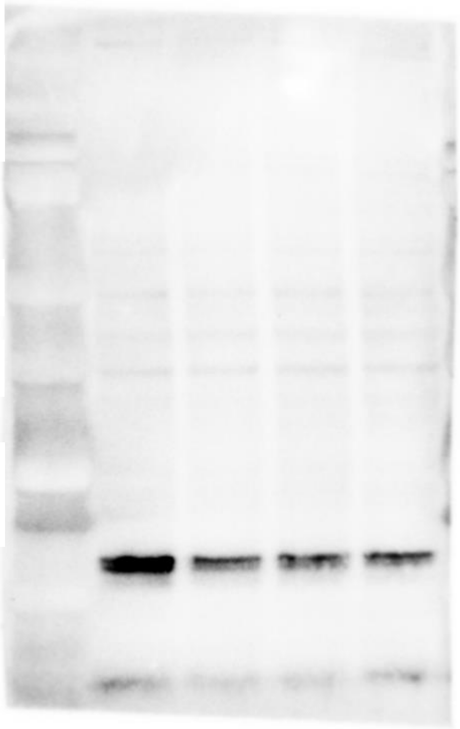

c-MYC

MW
250
150
100
75
50
37
25
20
15
10

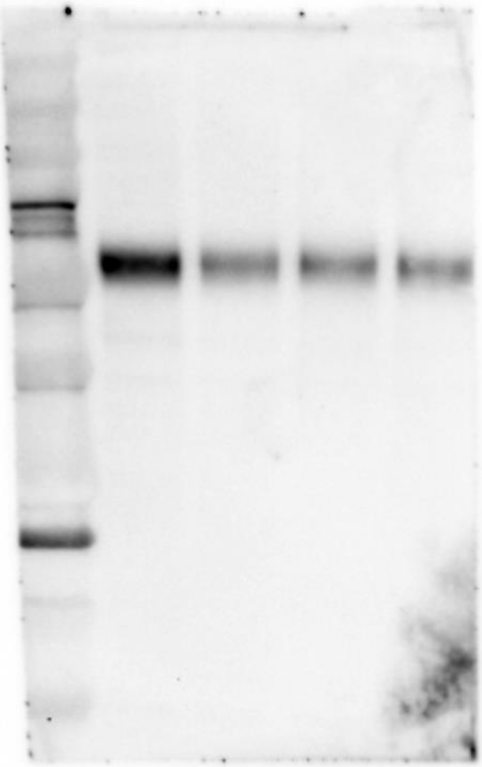

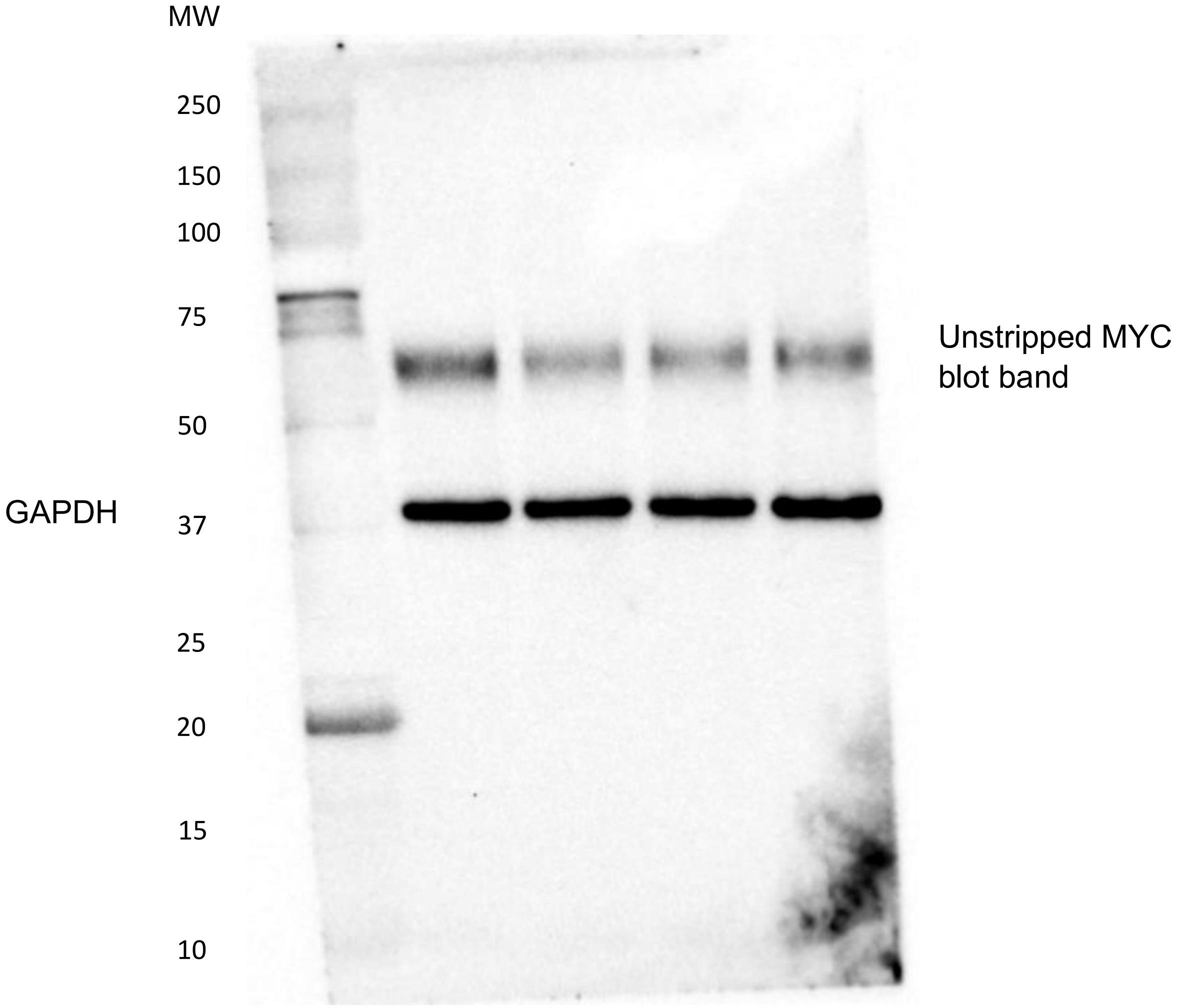
MW
250
150
100
75
50
37
25
20
15
10
GAPDH
Unstripped MYC blot band
