# Supplementary material for: Mechanism-centric regulatory network identifies NME2 and MYC programs as markers of Enzalutamide resistance in CRPC
Source: Nat Commun. 2024 Jan 8;15:352. doi: 10.1038/s41467-024-44686-5 (PMC10774320; doi:10.1038/s41467-024-44686-5)
Supplement: Supplementary file 3 — Description of Additional Supplementary Files [file 41467_2024_44686_MOESM3_ESM.pdf]

Description of Additional Supplementary files

Supplementary Data 1. Description of the datasets

Supplementary Data 2. Common target genes of MYC pathway and AR transcriptional regulatory program

Supplementary Data 3. Network reconstructed using SU2C East Coast cohort, showing edges with FDR p-value < 0.05 (estimated through bootstrap analysis) and edge weights = 100

Supplementary Data 4. Gene expression signatures and corresponding pathway and transcriptional regulatory analyses for comparing intact, Enzalutamide sensitive, and Enzalutamide resistant conditions.

Supplementary Data 5. Prioritization of transcriptional regulatory programs affecting MYC pathway.

Supplementary Data 6. Common target genes of MYC pathway and NME2 transcriptional regulatory program

Supplementary Data 7. Comparison of MYC and NME2 programs to transcriptomic and genomic markers of prostate cancer aggressiveness, response to ADT and ARSIs, and response to Enzalutamide.
