# Supplementary material for: Mechanism-centric regulatory network identifies NME2 and MYC programs as markers of Enzalutamide resistance in CRPC
Source: Nat Commun. 2024 Jan 8;15:352. doi: 10.1038/s41467-024-44686-5 (PMC10774320; doi:10.1038/s41467-024-44686-5)
Supplement: Supplementary file 5 — Reporting Summary [file 41467_2024_44686_MOESM5_ESM.pdf]

nature portfolio

Corresponding author(s): Antonina Mitrofanova
Sarki A. Abdulkadir
Vishal Kothari

Last updated by author(s): 12/13/2023

Reporting Summary

Nature Portfolio wishes to improve the reproducibility of the work that we publish. This form provides structure for consistency and transparency in reporting. For further information on Nature Portfolio policies, see our Editorial Policies and the Editorial Policy Checklist.

Statistics

For all statistical analyses, confirm that the following items are present in the figure legend, table legend, main text, or Methods section.

n/a	Confirmed	
☐	☒	The exact sample size (*n*) for each experimental group/condition, given as a discrete number and unit of measurement
☐	☒	A statement on whether measurements were taken from distinct samples or whether the same sample was measured repeatedly
☐	☒	The statistical test(s) used AND whether they are one- or two-sided<br>*Only common tests should be described solely by name; describe more complex techniques in the Methods section.*
☐	☒	A description of all covariates tested
☐	☒	A description of any assumptions or corrections, such as tests of normality and adjustment for multiple comparisons
☐	☒	A full description of the statistical parameters including central tendency (e.g. means) or other basic estimates (e.g. regression coefficient) AND variation (e.g. standard deviation) or associated estimates of uncertainty (e.g. confidence intervals)
☐	☒	For null hypothesis testing, the test statistic (e.g. *F*, *t*, *r*) with confidence intervals, effect sizes, degrees of freedom and *P* value noted<br>*Give P values as exact values whenever suitable.*
☒	☐	For Bayesian analysis, information on the choice of priors and Markov chain Monte Carlo settings
☐	☒	For hierarchical and complex designs, identification of the appropriate level for tests and full reporting of outcomes
☐	☒	Estimates of effect sizes (e.g. Cohen's *d*, Pearson's *r*), indicating how they were calculated

Our web collection on statistics for biologists contains articles on many of the points above.

Software and code

Policy information about availability of computer code

Data collection	Code used by the manuscript has been made available as a package TR2PATH in GitHub (https://github.com/mitrofanova-lab/TR2PATH)
Data analysis	All codes used in the manuscript has been made available as a package TR2PATH in GitHub (https://github.com/mitrofanova-lab/TR2PATH)

For manuscripts utilizing custom algorithms or software that are central to the research but not yet described in published literature, software must be made available to editors and reviewers. We strongly encourage code deposition in a community repository (e.g. GitHub). See the Nature Portfolio guidelines for submitting code & software for further information.

Data

Policy information about availability of data

All manuscripts must include a data availability statement. This statement should provide the following information, where applicable:

- Accession codes, unique identifiers, or web links for publicly available datasets
- A description of any restrictions on data availability
- For clinical datasets or third party data, please ensure that the statement adheres to our policy

The mechanism-centric CRPC-specific network is available in Supplementary Data 2. Data supporting the findings of this study were obtained from (a detailed description of the datasets is available in Supplementary Data 1):
(i) dbGaP:
1. Stand Up To Cancer East Coast Prostate Cancer cohort, RNA-sequencing data, phs000915.v2.p2 [https://www.ncbi.nlm.nih.gov/projects/gap/cgi-bin/study.cgi?

study_id=phs000915.v2.p2]. Access could be obtained through dbGap portal.
2. PROMOTE Prostate Cancer cohort, RNA-sequencing data, phs001141.v1.p1 [https://www.ncbi.nlm.nih.gov/projects/gap/cgi-bin/study.cgi?study_id=phs001141.v1.p1]. Access could be obtained through dbGap portal.
3. Beltran et al, RNA-sequencing data, phs000909.v1.p1 [https://www.ncbi.nlm.nih.gov/projects/gap/cgi-bin/study.cgi?study_id=phs000909.v1.p1]. Access could be obtained through dbGap portal.
4. SU2C West Coast Prostate Cancer cohort, RNA-sequencing data, phs001648.v2.p1 [https://www.ncbi.nlm.nih.gov/projects/gap/cgi-bin/study.cgi?study_id=phs001648.v2.p1], downloaded from [https://portal.gdc.cancer.gov/projects/WCDT-MCRPC]. Access could be obtained through dbGap portal.

(ii) cBioPortal:
1. Abida et al, RNA-sequencing data [https://github.com/cBioPortal/datahub/tree/master/public/prad_su2c_2019]. Data could be downloaded directly from cBioPortal.

(iii) GEO:
1. Kregel et al, microarray gene expression data, GSE78201 [https://www.ncbi.nlm.nih.gov/geo/query/acc.cgi?acc=GSE78201]. Data could be downloaded directly from GEO.
2. Kumar et al, microarray gene expression data, GSE77930 [https://www.ncbi.nlm.nih.gov/geo/query/acc.cgi?acc=GSE77930]. Data could be downloaded directly from GEO.

(iv) Broad Single Cell Portal:
1. He et al., RNA-sequencing data [https://singlecell.broadinstitute.org/single_cell/study/SCP1244/transcriptional-mediators-of-treatment-resistance-in-lethal-prostate-cancer]. Data could be downloaded directly from Broad Single Cell Portal.

(v) Manuscript Supplemental Information
1. Alumkal et al., RNA-sequencing data: https://www.pnas.org/doi/10.1073/pnas.1922207117. Data could be obtained from the Supplementary Information in12.

Hallmark and C2 pathway gene sets were obtained from the molecular signatures database (MSigDB 3.0) https://www.gsea-msigdb.org/gsea/msigdb. Source data are provided with this paper: experimental source data are provided as a Source Data file and computational source data are provided as a part of TR-2-PATH R software package (https://github.com/mitrofanova-lab/TR2PATH) under DOI:10.5281/zenodo.10368948.

Research involving human participants, their data, or biological material

Policy information about studies with human participants or human data. See also policy information about sex, gender (identity/presentation), and sexual orientation and race, ethnicity and racism.

Reporting on sex and gender	NA
Reporting on race, ethnicity, or other socially relevant groupings	NA
Population characteristics	NA
Recruitment	NA
Ethics oversight	NA

Note that full information on the approval of the study protocol must also be provided in the manuscript.

Field-specific reporting

Please select the one below that is the best fit for your research. If you are not sure, read the appropriate sections before making your selection.

☒ Life sciences ☐ Behavioural & social sciences ☐ Ecological, evolutionary & environmental sciences

For a reference copy of the document with all sections, see nature.com/documents/nr-reporting-summary-flat.pdf

Life sciences study design

All studies must disclose on these points even when the disclosure is negative.

Sample size	Experiments were repeated at least three times, which allowed for performance of statistical tests (i.e., t-test, ANOVA etc.) that require standard deviation calculation and to achieve sufficient statistical power
Data exclusions	No data were excluded
Replication	Experimental methods were repeated at least 3 times to assess reproducibility and to achieve sufficient statistical power.
Randomization	For the in vivo experiment, mice were allocated to experimental arms based on cages, therefore all 4 or 5 mice from one cage received the

Randomization	same treatment (be it Doxycycline-laced water or normal water, together with either DMSO or Enzalutamide ip injection). Each cage was randomly assigned to one of the 4 treatment arms before the experiment start.
Blinding	Blinding was not performed for the in vivo study because of personnel and time constraint. We aimed for the same person to collect tumor size data via caliper throughout to ensure consistency and that same person was also the one administering the treatments.

Reporting for specific materials, systems and methods

We require information from authors about some types of materials, experimental systems and methods used in many studies. Here, indicate whether each material, system or method listed is relevant to your study. If you are not sure if a list item applies to your research, read the appropriate section before selecting a response.

Materials & experimental systems

n/a	Involved in the study	
☐	☒	Antibodies
☐	☒	Eukaryotic cell lines
☒	☐	Palaeontology and archaeology
☐	☒	Animals and other organisms
☒	☐	Clinical data
☒	☐	Dual use research of concern
☒	☐	Plants

Methods

n/a	Involved in the study	
☒	☐	ChIP-seq
☒	☐	Flow cytometry
☒	☐	MRI-based neuroimaging

Antibodies

Antibodies used	The following primary antibodies were used: c-Myc (Y69) (Abcam #ab32072, 1:1000 dilution), NME2 (4G7A8) (Abcam #ab204958, 1:1000 dilution), GAPDH (Cell Signaling Technology #3683, 1:5000 dilution), Actin (Cell Signaling Technology #5125S, 1:5000 dilution). For secondary antibodies, anti-rabbit (Bio-Rad #170-6515) or anti-mouse (Bio-Rad #170-6515) IgG (H+L)-HRP conjugate were used.
Validation	Abcam states that the primary c-Myc and NME2 antibodies were validated in WB, IHC and tested in human, as well as being knockout validated. The loading control antibodies from CST (Actin and GAPDH) are also validated (for WB and also KO validation) by the manufacturer and are each used in multiple citations.

Eukaryotic cell lines

Policy information about cell lines and Sex and Gender in Research

Cell line source(s)	LNCaP cells (clone FCG) and C42B cells were purchased from ATCC. LNCaP cells were originally derived from the lymph node of a 50-year old white male with confirmed metastatic prostate carcinoma. C42B cell line is a derivative of LNCaP cells. LNCaP and C42B EnzaRes cells were generated in house using the ATCC source cells. NME2 KO and shNME2 cells were also generated from the ATCC source cells.
Authentication	Cell lines have been authenticated by the vendor (ATCC) using STR profilling.
Mycoplasma contamination	We have performed routine Mycoplasma testing on all cell lines employed in the manuscript and confirmed they are negative.
Commonly misidentified lines (See ICLAC register)	We are not using commonly misidentified cell lines.

Animals and other research organisms

Policy information about studies involving animals; ARRIVE guidelines recommended for reporting animal research, and Sex and Gender in Research

Laboratory animals	We used FVB mice obtained from Jackson Laboratory (strain #001-800). All the experiments were performed with mice of age 6-8 weeks. Information on housing conditions, dark/light cycle, temperature and humidity are provided in the methods section of the manuscript.
Wild animals	No wild animals were used in the study.
Reporting on sex	All studies in the manuscript pertain to Castration Resistant Prostate Cancer and therefore apply only to animals of male sex. We have not used or considered using female sexed animals because they do not have sufficient circulating androgen levels to support prostate cancer xenograft growth.
Field-collected samples	No field collected samples were used in the study

Ethics oversight | All animal experiments and procedures were performed in compliance with ethical standards and the approval of the Northwestern University Animal Care and Use Committee (IACUC).

Note that full information on the approval of the study protocol must also be provided in the manuscript.

Plants

Seed stocks | NA

Novel plant genotypes | NA

Authentication | NA
